# The IL-33:ST2 axis is unlikely to play a central fibrogenic role in idiopathic pulmonary fibrosis

**DOI:** 10.1186/s12931-023-02334-4

**Published:** 2023-03-23

**Authors:** Katherine E. Stephenson, Joanne Porte, Aoife Kelly, William A. Wallace, Catherine E. Huntington, Catherine L. Overed-Sayer, E. Suzanne Cohen, R. Gisli Jenkins, Alison E. John

**Affiliations:** 1 Division of Respiratory Medicine, School of Medicine, University of Nottingham, Nottingham, UK; 2 Bioscience Asthma and Skin Immunity, Research and Early Development, Respiratory and Immunology, BioPharmaceuticals R&D, AstraZeneca, Cambridge, UK; 3 Division of Pathology, University of Edinburgh, Edinburgh, UK; 4 Biologics Engineering, R&D, AstraZeneca, Cambridge, UK; 5 Bioscience COPD/IPF, Research and Early Development, Respiratory and Immunology, BioPharmaceuticals R&D, AstraZeneca, Cambridge, UK; 6 National Heart and Lung Institute, Imperial College London, London, UK; 7 Margaret Turner Warwick Centre for Fibrosing Lung Disease, Imperial College London, London, UK; 8 Interstitial lung disease unit, Royal Brompton Hospital, London, UK

**Keywords:** Idiopathic pulmonary fibrosis, IL-33, ST2, Fibroblasts, Bleomycin-induced pulmonary fibrosis, Precision-cut lung slices

## Abstract

**Background:**

Idiopathic pulmonary fibrosis (IPF) is a devastating interstitial lung disease (ILD) with limited treatment options. Interleukin-33 (IL-33) is proposed to play a role in the development of IPF however the exclusive use of prophylactic dosing regimens means that the therapeutic benefit of targeting this cytokine in IPF is unclear.

**Methods:**

IL-33 expression was assessed in ILD lung sections and human lung fibroblasts (HLFs) by immunohistochemistry and gene/protein expression and responses of HLFs to IL-33 stimulation measured by qPCR. In vivo, the fibrotic potential of IL-33:ST2 signalling was assessed using a murine model of bleomycin (BLM)-induced pulmonary fibrosis and therapeutic dosing with an ST2-Fc fusion protein. Lung and bronchoalveolar lavage fluid were collected for measurement of inflammatory and fibrotic endpoints. Human precision-cut lung slices (PCLS) were stimulated with transforming growth factor-β (TGFβ) or IL-33 and fibrotic readouts assessed.

**Results:**

IL-33 was expressed by fibrotic fibroblasts in situ and was increased by TGFβ treatment in vitro. IL-33 treatment of HLFs did not induce *IL6*, *CXCL8*, *ACTA2* and *COL1A1* mRNA expression with these cells found to lack the IL-33 receptor ST2. Similarly, IL-33 stimulation had no effect on *ACTA2*, *COL1A1*, *FN1* and fibronectin expression by PCLS. Despite having effects on inflammation suggestive of target engagement, therapeutic dosing with the ST2-Fc fusion protein failed to reduce BLM-induced fibrosis measured by hydroxyproline content or Ashcroft score.

**Conclusions:**

Together these findings suggest the IL-33:ST2 axis does not play a central fibrogenic role in the lungs with therapeutic blockade of this pathway unlikely to surpass the current standard of care for IPF.

**Supplementary Information:**

The online version contains supplementary material available at 10.1186/s12931-023-02334-4.

## Background

Idiopathic pulmonary fibrosis (IPF) is a debilitating interstitial lung disease (ILD) characterised by the excessive deposition of extracellular matrix by fibroblasts and an irreversible loss of lung function [1]. With a median survival of approximately 3 years from diagnosis, the prognosis for patients with IPF is worse than many types of cancer [2]. Despite its incidence continuing to increase, the aetiology and underlying pathophysiology of IPF remains unclear [1]. Although two drugs are currently licensed to treat IPF, their failure to halt disease progression and use-limiting side effects [3] demonstrate an urgent need to better understand the pathogenesis of IPF and identify new therapeutic targets.

The IL-1 family cytokine interleukin-33 (IL-33) is stored in the nucleus of multiple cell types and is released following cell damage or death [4–7]. Upon its release, IL-33 can interact with its transmembrane receptor ST2L (also known as serum stimulated-2 (ST2) or Interleukin-1 Receptor Like 1 (*IL1RL1*)) or be neutralised by its decoy receptor soluble ST2 (sST2) [8]. ST2-dependent signalling can initiate inflammation and promote wound healing following tissue damage [9]. Despite playing an important homeostatic role, dysregulation of the IL-33:ST2 axis has been implicated in the pathogenesis of several diseases including IPF [9, 10]. Indeed, high levels of IL-33 have been observed in the bronchoalveolar lavage fluid (BALF) [11], exhaled breath condensate [12] and lung tissue [13, 14] of IPF patients, with elevated concentrations of sST2 measured in IPF serum during exacerbations [15]. Additionally, IL-33 has been implicated in the bleomycin (BLM) mouse model of pulmonary fibrosis with germline ST2 deletion, IL-33 neutralising antibodies and sST2 all leading to reduced lung fibrosis in vivo [16–19]. Since IL-33 overexpression and treatment potentiates BLM-induced fibrosis [14, 16], IL-33 has been proposed to act as a key pro-fibrotic mediator during the development of IPF. However, since IL-33 signalling has only ever been modulated during the inflammatory phase of the BLM mouse model [14, 16–18], it is possible that these results reflect the importance of IL-33 during inflammation and repair rather than in fibrosis. Consequently, the therapeutic benefit of targeting the IL-33:ST2 axis in IPF is unclear.

Transforming growth factor-β (TGFβ) is a key pro-fibrotic cytokine that induces the production of extracellular matrix by primary human lung fibroblasts (HLFs) in vitro [20, 21]. In vivo, TGFβ plays an important role in pulmonary fibrosis with therapies targeting TGFβ activation and signalling shown to reduce fibrosis in multiple model systems [22–24]. Moreover, TGFβ can induce fibrotic changes in human precision-cut lung slices (PCLS) [25] and explanted lung parenchymal tissue samples [26].

To understand the role of IL-33 in established pulmonary fibrosis, we assessed the localisation of IL-33 in fibrotic lung tissue and determined whether IL-33 expression by HLFs could be regulated by TGFβ. Furthermore, the effects of blocking IL-33 signalling during the fibrotic phase of the BLM mouse model were established and the ability of IL-33 to induce fibrotic changes in human PCLS investigated.

## Methods

### IL-33 Immunohistochemistry (IHC)

Formalin fixed, paraffin embedded tissue samples from 43 ILD patients (patient characteristics previously reported in Saini et al. [27]) and 4 non-ILD controls (normal adjacent tissue from patients undergoing lung cancer resections) were obtained from the University of Edinburgh and the Nottingham Respiratory Research Unit following informed consent and local ethics approval by South East Scotland SAHSC Bioresource and the MRC Nottingham Molecular Pathology Node respectively. IHC was performed as previously described [27] using a mouse anti-IL-33 monoclonal antibody (Nessy-1, Abcam, Cambridge, UK).

### Cell culture and treatment

Non-IPF and IPF patient-derived primary human lung fibroblasts (HLFs) were obtained from the Nottingham Respiratory Research Unit and cultured as previously described [28]. Following 24 h serum-starvation, passage 6 HLFs were stimulated with 2 ng/ml TGFβ or 10 ng/ml IL-33 (R&D Systems, Abingdon, UK) with supernatants, RNA and protein collected as previously described [29].

### Bleomycin (BLM) mouse model

Animal studies using male C57BL/6 mice were approved by the University of Nottingham Animal Welfare and Ethical Review Board, carried out in accordance with Animals (Scientific Procedures) Act 1986 and planned and reported in compliance with the ARRIVE (Animal Research: Reporting of In Vivo Experiments) guidelines [30]. Pulmonary fibrosis was induced with 60 IU BLM sulphate (Kyowa Kirrin, Slough, UK) as previously described [24] and mice dosed every 72 h from day 14–28 with 10 mg/kg of ST2-Fc fusion protein (AstraZeneca, Cambridge, UK) via intraperitoneal (i.p.) injection. Upon completion of the study, mice were randomly allocated into groups for either biochemical or histological analysis.

For mice in the biochemical analysis group, bronchoalveolar lavage (BAL) was collected and lung tissue snap frozen as previously described [24]. Total BAL cell counts were performed using a hemacytometer. For differential cell counts, 200 μl of BAL was cytospun onto glass slides and stained using a Diff-Quick staining kit (Siemens Healthineers, Camberley, UK) according to the manufacturer’s instructions. Differential cell counts were performed as previously described [24] with the remaining BAL fluid (BALF) collected for analysis by ELISA. Lung tissue was ground into a powder using liquid nitrogen. Lung hydroxyproline content per lung set was measured as described previously [29]. RNA and protein were extracted as previously described [24, 29].

For mice assigned to the histological analysis group, the tracheas of these animals were cannulated and the pulmonary vasculature perfused with 40 U/ml of heparinised PBS (Sigma-Aldrich, St. Louis, MO, USA). Lungs were inflated with 10% formalin (VWR, Lutterworth, UK) under constant gravitational pressure (20 cm H_2_O) prior to removal and fixation in 10% formalin. All tissue was wax embedded and 5 μm thick sections cut for histological analysis. Tissue was dewaxed in xylene and rehydrated in graded ethanol. Sections were subsequently stained with Mayer’s haematoxylin and eosin or Weigert’s haematoxylin and Sirius red. Masson’s trichome staining was performed as previously described [29]. Stained sections were visualised using CaseViewer software (3DHISTECH, Budapest, Hungary) and the severity of lung fibrosis quantified by Ashcroft Scoring [31].

### ELISA analysis

Human (DY3625B) and mouse (DY3626) IL-33 and mouse ST2 (DY1004) ELISAs (R&D systems) were performed according to the manufacturer’s instructions.

### Precision-cut lung slices (PCLS)

Normal adjacent lung tissue (NAT) and IPF lung tissue was obtained from the Royal Papworth Hospital Research Tissue Bank with written consent and study approval from the NRES Committee East of England. Patient characteristics for lung samples used in PCLS experiments are summarised in Table 1.

**Table 1:** Patient characteristics for PCLS lung samples

	Non-IPF (n = 4)	IPF (n = 2)
Age, years	73 (67–76) (n = 3)	61 (56–66)
Gender, male/female	1/2 (n = 3)	2/0
Smoking status, ever/never-smokers	3/0 (n = 3)	1/1
FVC % predicted, %	117.6 (109.8–121.0) (n = 3)	46.5 (37.0–56.0)

Data presented as median (IQR) unless specified. Clinical data for one non-IPF donor not available. *IPF* idiopathic pulmonary fibrosis, *IQR* interquartile range, *FVC* forced vital capacity, *n* number of available data

PCLS were generated as previously described [32]. Once generated, PCLS were rested for 4 days in Small Airway Epithelial Cell Media supplemented with 2.5 mg/ml Bovine Serum Albumin, 0.004 ml/ml Bovine Pituitary Extract, 10 ng/ml EGF, 5 μg/ml Insulin, 0.5 μg/ml Hydrocortisone, 0.5 μg/ml Epinephrine, 6.7 ng/ml Triiodo-L-thyronine, 10 μg/ml Transferrin and 0.1 ng/ml Retinoic Acid (PromoCell, Heidelberg, Germany). Daily media changes were performed prior to stimulation every 24 h with 2 ng/ml TGFβ (R&D Systems) or 30 ng/ml IL-33 (Viva Biotech, Shanghai, China). PCLS (3–4/treatment) were pooled and processed for RNA or protein on day 7.

For RNA samples, PCLS were submerged in RNAlater (Sigma-Aldrich) for 24 h at 4 °C then stored at -80 °C prior to RNA isolation. To extract RNA from PCLS, 600 μl of TRI Reagent (Zymo Research, Irvine, CA, USA) was added to each thawed sample. Using a Retsch TissueLyser II and 5 mm stainless steel beads (Qiagen, Germantown, MD, USA), samples were homogenized at 20 Hz for 2 × 2 min. After centrifugation at 16,000 × g for 30 s to remove debris, RNA was isolated using the Direct-zol™ RNA Miniprep kit according to the manufacturer’s instructions.

For protein samples, 150 μl of Cell Lysis Buffer (Cell Signalling Technology, Danvers, MA, USA) supplemented with Halt™ Protease and Phosphatase Inhibitor Cocktail (Thermo Fisher Scientific, Loughborough, UK) was added and samples stored at -80 °C. Thawed samples were homogenized using an OMNI Tissue Homogenizer with protein lysates clarified and quantified as previously described [24].

### Gene expression analysis

RNA from cells, murine tissue and human PCLS was converted into cDNA using Moloney murine leukemia virus reverse transcriptase (Promega, Madison, WI, USA), SuperScript® IV reverse transcriptase (Invitrogen, Carlsbad, CA, USA) and High-Capacity RNA-to-cDNA™ (Applied Biosystems, Vilnius, Lithuania) kits respectively. All reverse transcription reactions were performed according to the appropriate manufacturer’s instructions. cDNA from cells and murine lung tissue was analysed by qPCR using oligonucleotide primers (Eurofins, Luxemburg), KAPA SYBR® FAST qPCR master mix (Roche, Basel, Switzerland) and a MxPro3005 QPCR system (Agilent Technologies, Manchester, UK). All reactions were performed using the following program: initial denaturation at 95 °C for 30 s followed by 40 cycles of 95 °C for 5 s, 60 °C for 30 s and 72 °C for 15 s. Amplification of a single DNA product was confirmed by melting curve analysis. The following primer sequences were used for human (h) and mouse (m): h*ACTA2* forward 5’-TGTGCTGGACTCTGGAGATG-3’, reverse 5’-GACAATCTCACGCTCAGCAG-3’; h*β2M* forward 5’-AATCCAAATGCGGCATCT-3’, reverse 5’-GAGTATGCCTGCCGTGTG-3’; h*COL1A1* forward 5’-CCAGCAAATGTTCCTTTTTG-3’, reverse 5’-AAAATTCACAAGTCCCCATC-3’; h*CXCL8* forward 5’-ATGACTTCCAAGCTGGCCGTGGCT-3’, reverse 5’-TCTCAGCCCTCTTCAAAAACTTCTC-3’; h*IL6* forward 5’-CAATAACCACCCCTGACCCA-3’, reverse 5’-GCGCAGAATGAGATGAGTTGTC-3’; h*IL33* forward 5’-CCTGTCAACAGCAGTCTACT-3’, reverse 5’-TTGGCATGCAACCAGAAGTC-3’; m*HPRT* forward 5’-CCAGCAGGTCAGCAAAGAACT-3’, reverse 5’-TGAAAGACTTGCTCGAGATGTCA-3’ and m*IL33* forward 5’-CACATTGAGCATCCAAGGAA-3’, reverse 5’-AACAGATTGGTCATTGTATGTACTCAG-3’. cDNA from PCLS was analysed by qPCR using TaqMan™ Assays, TaqMan™ Fast Advanced Master Mix and a 7900HT Fast Real-Time PCR System (Applied Biosystems). The following TaqMan™ Assays were purchased from Applied Biosystems: *ACTA2* (Hs00426835_g1), *β2M* (Hs00187842_m1), *COL1A1* (Hs00164004_m1) and *FN1* (Hs01549976_m1) with all reactions performed as follows: initial denaturation at 95 °C for 20 s followed by 40 cycles of 95 °C for 1 s and 60 °C for 20 s. Using MxPro qPCR software (Agilent Technologies) or SDS 2.4 software (Applied Biosystems), cycle threshold (Ct) values for both the target and housekeeping genes (human β2-microglobulin (*β2M*) and murine *HPRT*) were determined. All data was analysed using the ΔΔCT method with the expression of each gene of interest, relative to a housekeeper, calculated and presented as a fold change versus the stated control condition/group.

### Western blotting

Cell lysates were loaded into 12.5% bis/acrylamide (w/v) gels as previously described [29]. PCLS protein lysates were loaded into Bolt™ 4–12% gels (Invitrogen). Western blotting was performed and analysed as previously described [29, 33]. Primary antibodies used for western blotting included goat anti-IL-33 (AF3625; R&D systems), goat anti-ST2 (AF523; R&D systems), mouse anti-α-Tubulin (TU-02; Santa Cruz Biotechnology, Santa Cruz, CA, USA), rabbit anti-Fibronectin (ab2413; Abcam) and rabbit anti-GAPDH (ab181603; Abcam). Anti-goat HRP-conjugated secondary antibody (HAF109; R&D systems) and anti-rabbit (P0448) and anti-mouse (P0447) HRP-conjugated secondary antibodies (Dako, Glostrup, Denmark).

### Statistical analysis

Statistical analysis was performed using GraphPad Prism 8 with P values ≤ 0.05 considered statistically significant. The distribution of all data was determined by Shapiro–Wilk normality test. Data are reported as either mean (parametric) or median (non-parametric) with all individual data points shown where appropriate. For parametric data, unpaired and paired t-tests were used to determine statistical significance between two unmatched and matched groups whilst One- and Two-way (ordinary and repeated measures) ANOVAs were used to compare multiple (unpaired and paired) groups and variables. For non-parametric unpaired data, statistical significance between two or more groups was assessed by Mann–Whitney U test and Kruskal–Wallis test respectively. For non-parametric paired data, statistical significance between two or more groups was assessed by Wilcoxon signed-rank test and Friedman test respectively.

## Results

### IL-33 is expressed by fibroblasts in IPF

**Fig. 1:**
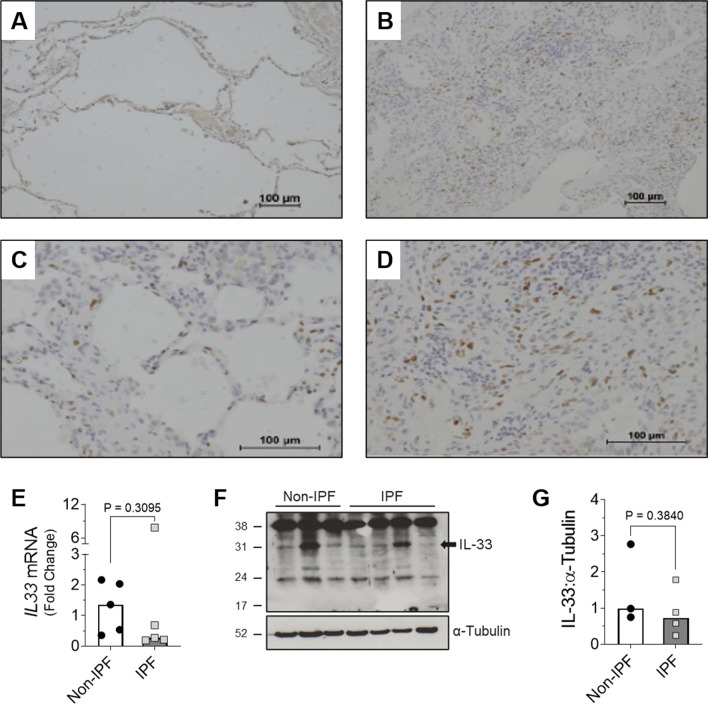
IL-33 expression in ILD lung tissue and non-IPF and IPF lung fibroblasts. IL-33 expressing cells in ILD (n = 43) and non-ILD (n = 4) lung tissue were identified via IHC. Representative images of IL-33 staining in non-ILD controls at × 10 magnification (**A**) and ILD samples at × 10 magnification (**B**) and × 20 magnification (**C**, **D**) are shown. Basal *IL33* gene expression in IPF (n = 5) and non-IPF (n = 5) human lung fibroblasts (HLFs) was measured by qPCR and normalised to the average ΔCt value calculated for non-IPF cells. Bars indicate median values and each data point represents a single donor. Statistical analysis was performed by Mann–Whitney U test (**E**). 20 μg protein/lane was separated by SDS-PAGE and basal IL-33 protein expression by non-IPF (n = 3) and IPF (n = 4) HLFs assessed by western blot (**F**) and quantified relative to α-Tubulin via densitometry (**G**). A representative cropped western blot is shown with the whole IL-33 blot viewable in Additional file 1: Fig. S1. Bars indicate mean values and each data point represents a single donor with statistical analysis performed by Unpaired t-test

To identify the cellular expression of IL-33, lung tissue from IPF patients and a small subset of nonspecific interstitial pneumonitis (NSIP) patients (collectively referred to as ILD tissue) was assessed by immunohistochemistry. Compared with staining in non-ILD controls (Fig. 1A), IL-33 positive cells appeared to be present in greater abundance in ILD lung sections (Fig. 1B), with the majority of staining localised to cell nuclei (Fig. 1C, D). Morphological analysis of the ILD tissue sections suggested that a number of the IL-33 positive cells were fibroblasts (Fig. 1C, D). To confirm that IPF fibroblasts express IL-33, we analysed IL-33 levels in fibroblasts from IPF and non-diseased controls (Fig. 1E–G). Both non-IPF and IPF primary human lung fibroblasts (HLFs) were found to express IL-33 mRNA (Fig. 1E) and protein (Fig. 1F, G) in vitro, although levels were variable with no significant difference observed between non-IPF and IPF cells.

### TGFβ increases IL-33 gene and protein expression by fibroblasts

**Fig. 2:**
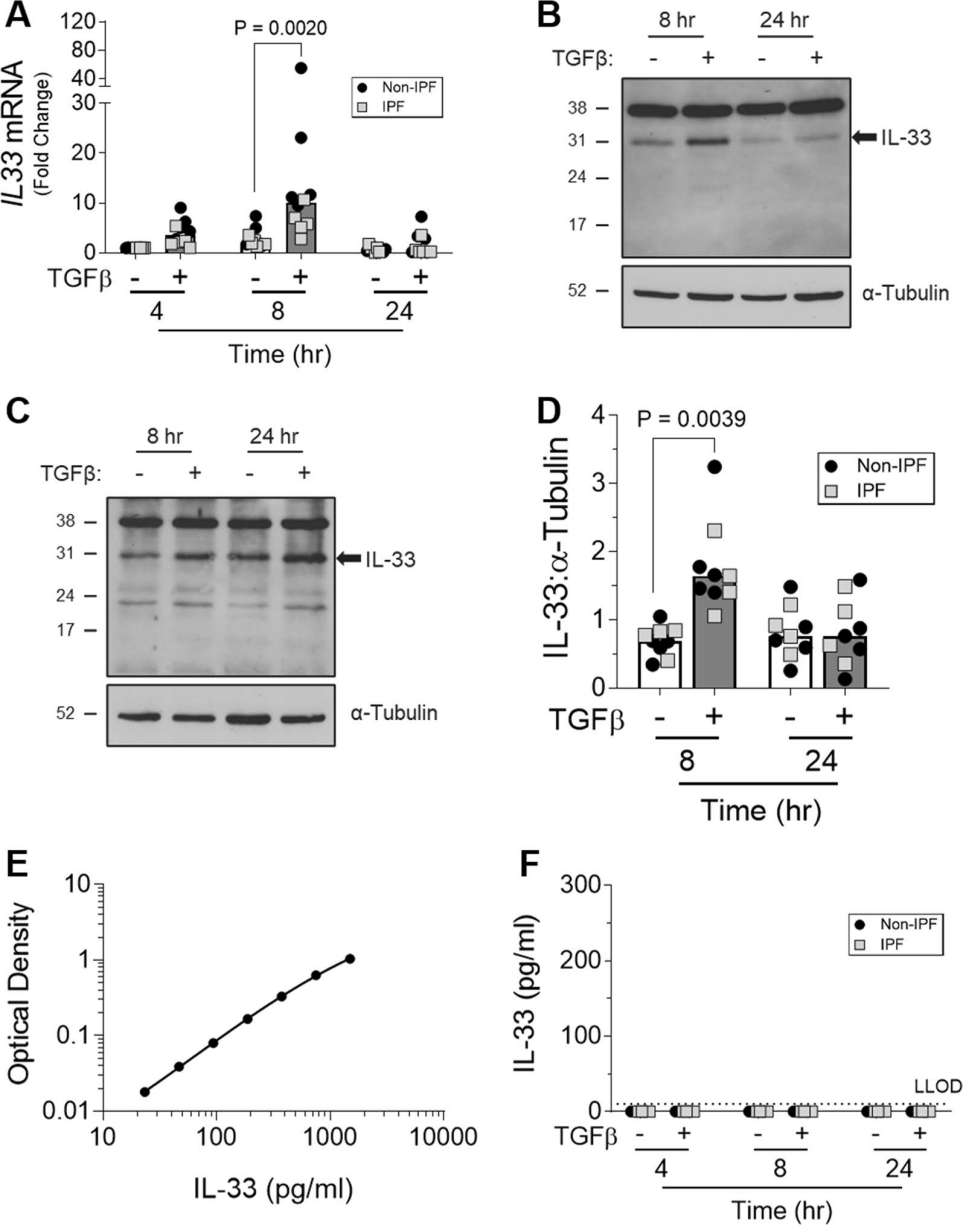
The effect of TGFβ on IL-33 expression by fibroblasts over time. Non-IPF (n = 5) and IPF (n = 5) HLFs were stimulated with 2 ng/ml of TGFβ for 4, 8 and 24 h and *IL33* gene expression measured by qPCR. For each donor, all data was normalised to the unstimulated media alone control at 4 h (**A**). Non-IPF (n = 5) and IPF (n = 4) HLFs were stimulated with 2 ng/ml of TGFβ for 8 and 24 h, total protein isolated and 20 μg protein/lane separated by SDS-PAGE. IL-33 protein expression by non-IPF (**B**) and IPF (**C**) HLFs was assessed by western blot and quantified relative to α-Tubulin via densitometry (**D**). Representative cropped western blots from non-IPF and IPF donors are shown. Whole IL-33 blots for the representative non-IPF and IPF donors are viewable in Additional file 1: Figs. S2 and Fig. S3 respectively. IL-33 release from non-IPF (n = 3) and IPF (n = 3) HLFs treated with 2 ng /ml TGFβ for 4, 8 or 24 h was measured by ELISA. Recombinant IL-33 was titrated as a positive control (**E**) and analysed alongside neat HLF supernatants (**F**) Non-IPF and IPF donors shown in black and grey respectively. Bars indicate median values and each data point represents a single donor with statistical analysis performed by Wilcoxon signed-rank test

To understand the effect of the pro-fibrotic environment on IL-33 expression by fibroblasts, diseased and control HLFs were stimulated with TGFβ. Stimulation with TGFβ increased *IL33* gene expression in both non-IPF and IPF HLFs after 4 h relative to unstimulated control cells. *IL33* expression peaked at 8 h before returning to baseline by 24 h (Fig. 2A). Similar changes were observed at the protein level for both non-IPF (Fig. 2B, D) and IPF (Fig. 2C, D) cells. Interestingly, despite detection of the recombinant IL-33 control (Fig. 2E), no secreted IL-33 could be measured in supernatants collected from TGFβ-treated HLFs at any time point tested (Fig. 2F). Furthermore, there were no clear differences in TGFβ-induced IL-33 levels between fibrotic or non-fibrotic HLFs (Fig. 2A, D).

### Extracellular IL-33:ST2 interactions do not promote pro-fibrotic activity in fibroblasts

**Fig. 3:**
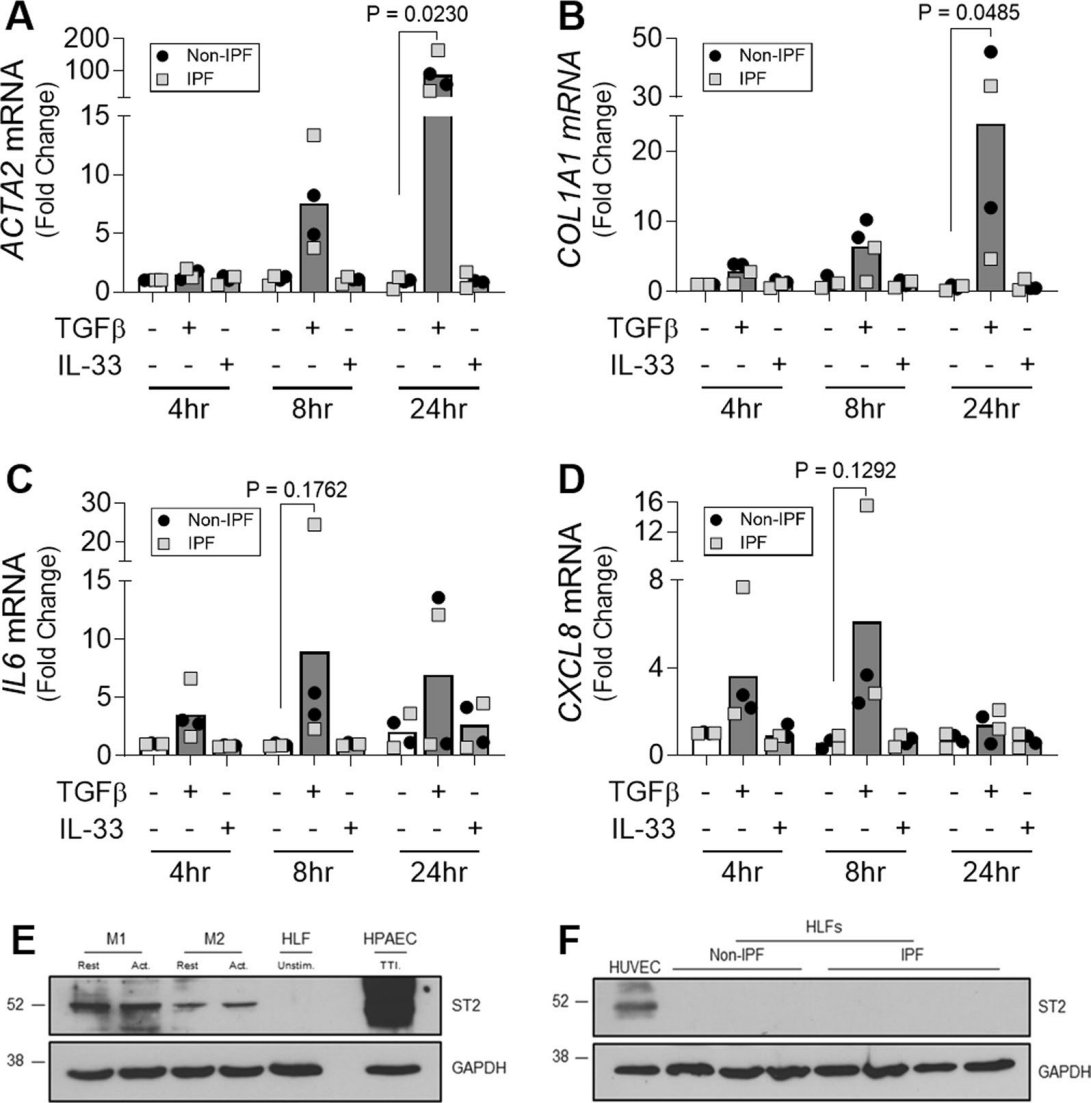
The effect of IL-33 on the pro-fibrotic activity of fibroblasts. Non-IPF (n = 2) and IPF (n = 2) HLFs were stimulated with 2 ng/ml of TGFβ or 10 ng/ml of IL-33 for 4, 8 and 24 h and *ACTA2* (**A**), *COL1A1* (**B**), *IL6* (**C**) and *CXCL8* (**D**) gene expression measured by qPCR. For each donor, all data was normalised to the unstimulated media alone control at 4 h. Non-IPF and IPF donors are shown in black and grey respectively. Bars indicate median values and each data point represents a single donor with statistical analysis performed by Wilcoxon signed-rank test. Whole cell lysates from resting (rest.) M1 and M2 MDMs, activated (act.) M1 (IFNγ stimulated) and M2 (IL-4 stimulated) MDMs, HPAECs stimulated with 5 ng/ml of TNFα, 5 ng/ml of TGFβ and 0.1 ng/ml of IL-1β (TTI) and a single non-IPF HLF donor were separated by SDS-PAGE (20 μg protein/lane) and expression of ST2 and GAPDH measured by western blot (**E**). Whole cell lysates from HUVECs, non-IPF (n = 3) and IPF (n = 4) HLFs were separated by SDS-PAGE (20 μg protein/lane) and the expression of ST2 and GAPDH determined by western blot (**F**). Representative cropped western blots are shown

To determine whether IL-33 had pro-fibrotic effects independent of TGFβ in vitro, pro-fibrotic and pro-inflammatory mediators were measured in response to TGFβ and IL-33 stimulation. As expected, TGFβ induced *ACTA2* and *COL1A1* expression in both non-IPF and IPF HLFs over time (Fig. 3A, B) with significant increases observed at 24 h versus time-matched unstimulated controls. In contrast, IL-33 had no effect on the expression of these genes at any time point (Fig. 3A, B). TGFβ also increased *IL6* and *CXCL8* expression by non-IPF and IPF HLFs whereas IL-33 had no effect on the expression of these genes (Fig. 3C, D).

To understand why HLFs were unable to respond to IL-33, expression of the IL-33 receptor, ST2, was assessed by western blotting. Although ST2 was detected in M1 and M2 monocyte-derived macrophages (MDMs), human pulmonary artery endothelial cells (HPAECs) and human umbilical vein endothelial cells (HUVECs), cell types known to respond to IL-33, no ST2 was detectable in HLFs (Fig. 3E, F).

### Therapeutic inhibition of IL-33 signalling does not reduce established bleomycin (BLM)-induced pulmonary fibrosis

**Fig. 4:**
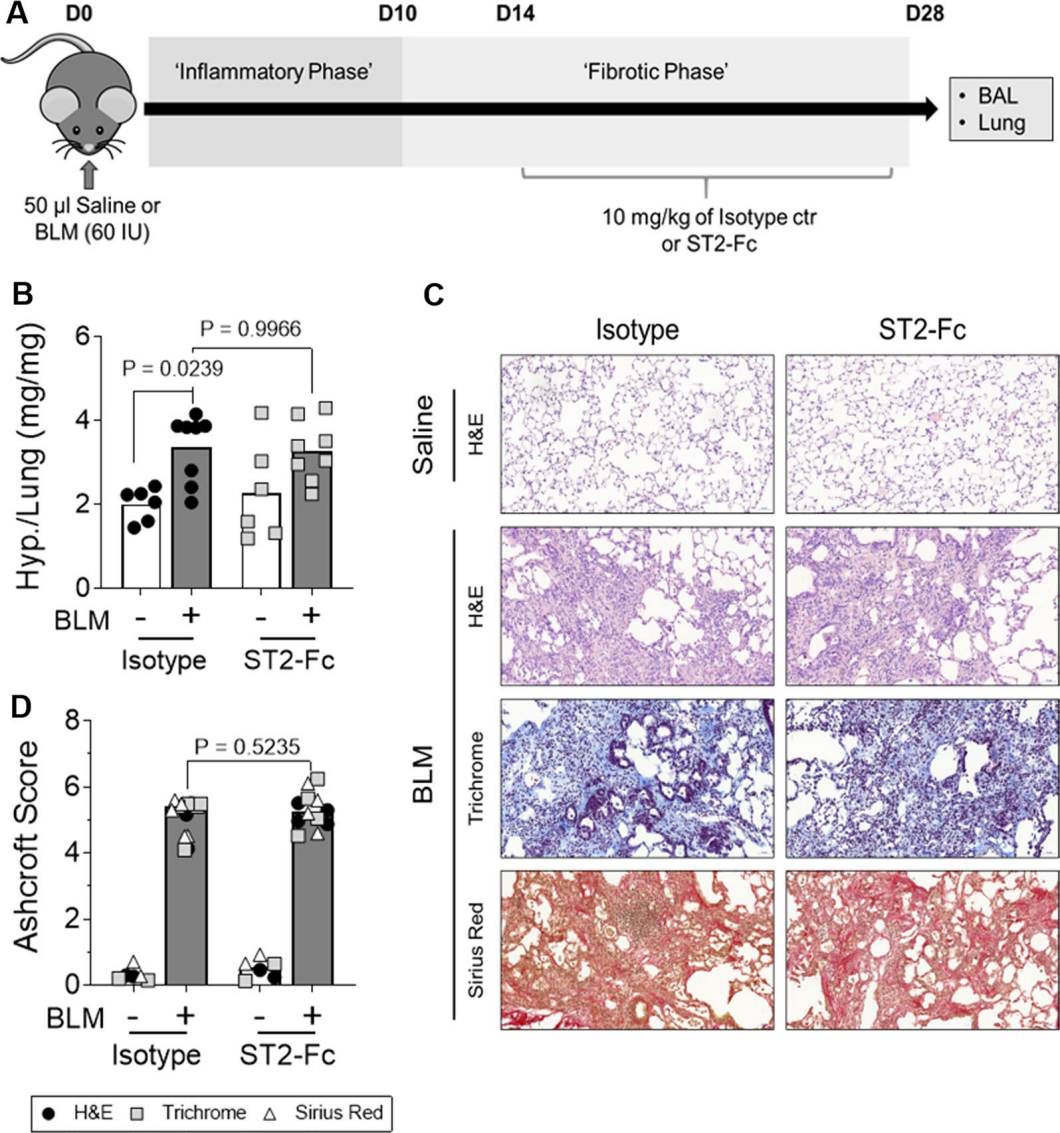
The effect of the ST2-Fc fusion protein on established BLM-induced pulmonary fibrosis. Saline or bleomycin (BLM) treated mice were dosed intraperitonially (i.p) with 10 mg/kg of either isotype control or ST2-Fc fusion protein every 72 h from day 14–28 (**A**). Hydroxyproline (Hyp.) content was measured in lung homogenates (**B**). Bars indicate mean values and each data point represents a single mouse. n = 6–8 per treatment group and statistical analysis was performed by Two-way ANOVA with Tukey’s multiple comparisons test. Immunohistochemical staining of lung sections with H&E, Sirius red and Masson’s trichrome were performed with representative, low magnification (× 10) images of stained lung tissue sections shown (**C**). The severity of BLM-induced fibrosis was quantified by Ashcroft scoring with the scores for H&E, Trichrome and Sirius Red stained sections shown in black, grey and white respectively (**D**). Bars indicate median values and each mouse is represented by 3 data points (one for each stain). n = 2–4 per treatment group. Statistical analysis was performed by Mann–Whitney U test

To investigate whether extracellular IL-33 has pro-fibrotic effects in a complex biological system, an inhibitor of IL-33 signalling, ST2-Fc fusion protein [33, 34], was delivered during the fibrotic phase of the BLM model of pulmonary fibrosis (Fig. 4A). Therapeutic dosing with the ST2-Fc fusion protein had no effect on BLM-induced fibrosis as assessed by lung hydroxyproline levels (Fig. 4B), lung morphology and collagen deposition in the lungs (Fig. 4C & 4D). Taken together, these data suggest that the IL-33:ST2 axis does not represent a central pro-fibrotic pathway in the BLM mouse model of pulmonary fibrosis.

**Fig. 5:**
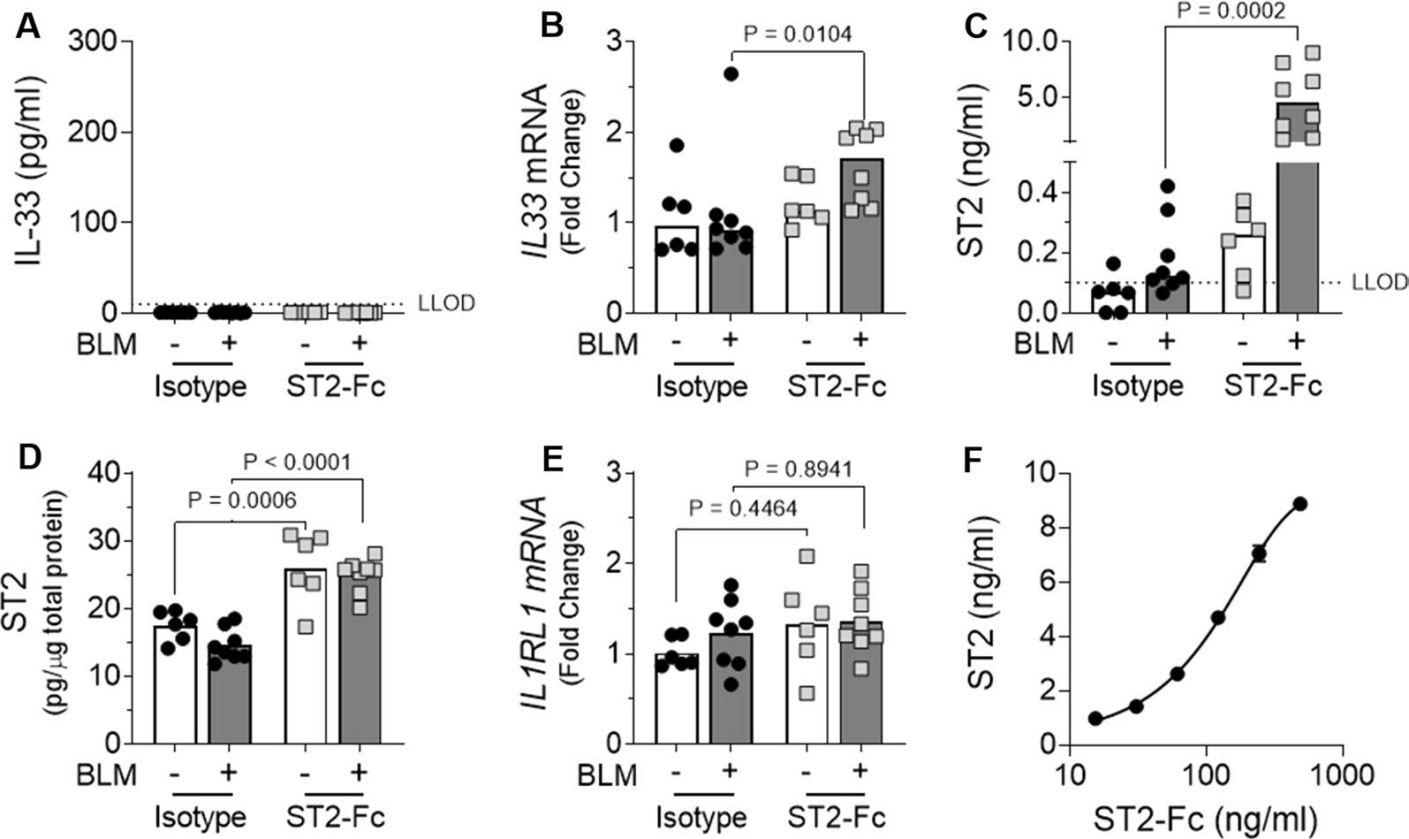
The effects of the ST2-Fc fusion protein on IL-33 and ST2 in vivo. ELISA analysis of IL-33 in the BALF (**A**). qPCR analysis of lung *IL33* mRNA levels with all values normalised to saline isotype control (**B**). ELISA analysis of ST2 levels in the BALF (**C**) and lung tissue (**D**). qPCR analysis of *IL1RL1* (ST2) gene expression in the lungs with all values normalised to saline-treated isotype control mice (**E**). Assessment of cross-reactivity between the ST2-Fc fusion protein and the ST2 ELISA kit (**F**). For Figures **B** & **D**, bars indicate median values and statistical analysis was performed by Mann–Whitney U test. For Figures **D** & **E** bars indicate mean values and statistical analysis was performed by Two-way ANOVA with Tukey’s multiple comparisons test. For **A**–**E**, each data point represents a single mouse. n = 6–8 per treatment group. For **F**, data is presented as mean ± SD

To ensure the ST2-Fc fusion protein was engaging its target and modulating IL-33 signalling, the effect of the fusion protein on IL-33 expression, ST2 levels and BLM-induced inflammation were assessed. BLM treated control mice that received isotype from day 14 to day 28 showed no detectable increase in IL-33 either at the protein level in the BALF nor at the gene level in lung tissue homogenates (Fig. 5A, B). In contrast, exposure to the ST2-Fc fusion protein significantly increased *IL33* gene expression in the lungs of BLM treated mice relative to those receiving isotype control (Fig. 5B).

As IL-33 can complex with soluble ST2 and prevent detection via the IL-33 ELISA, it was possible that changes in IL-33 could not be detected in the BALF due to the presence of high levels of sST2. To address this possibility, ST2 levels were measured in the BALF and lung tissue lysates on day 28 post BLM exposure. ST2 levels detected in BALF were increased in ST2-Fc treated mice compared with their respective isotype control groups, with a significant increase detected in BLM treated animals (Fig. 5C). As with the BALF samples, ST2 levels in lung homogenates were also highest in mice treated with ST2-Fc (Fig. 5D). Analysis of ST2 (*IL1RL1*) gene expression detected similar levels across all treatment groups and suggested that receptor expression was not modulated by treatment with ST2-Fc fusion protein or exposure to BLM (Fig. 5E). Since the ST2-Fc fusion protein could be directly detected by the ST2 ELISA kit (Fig. 5F), these results collectively suggest that the ST2-Fc fusion protein had reached the site of action in the lungs following i.p. delivery.

**Fig. 6:**
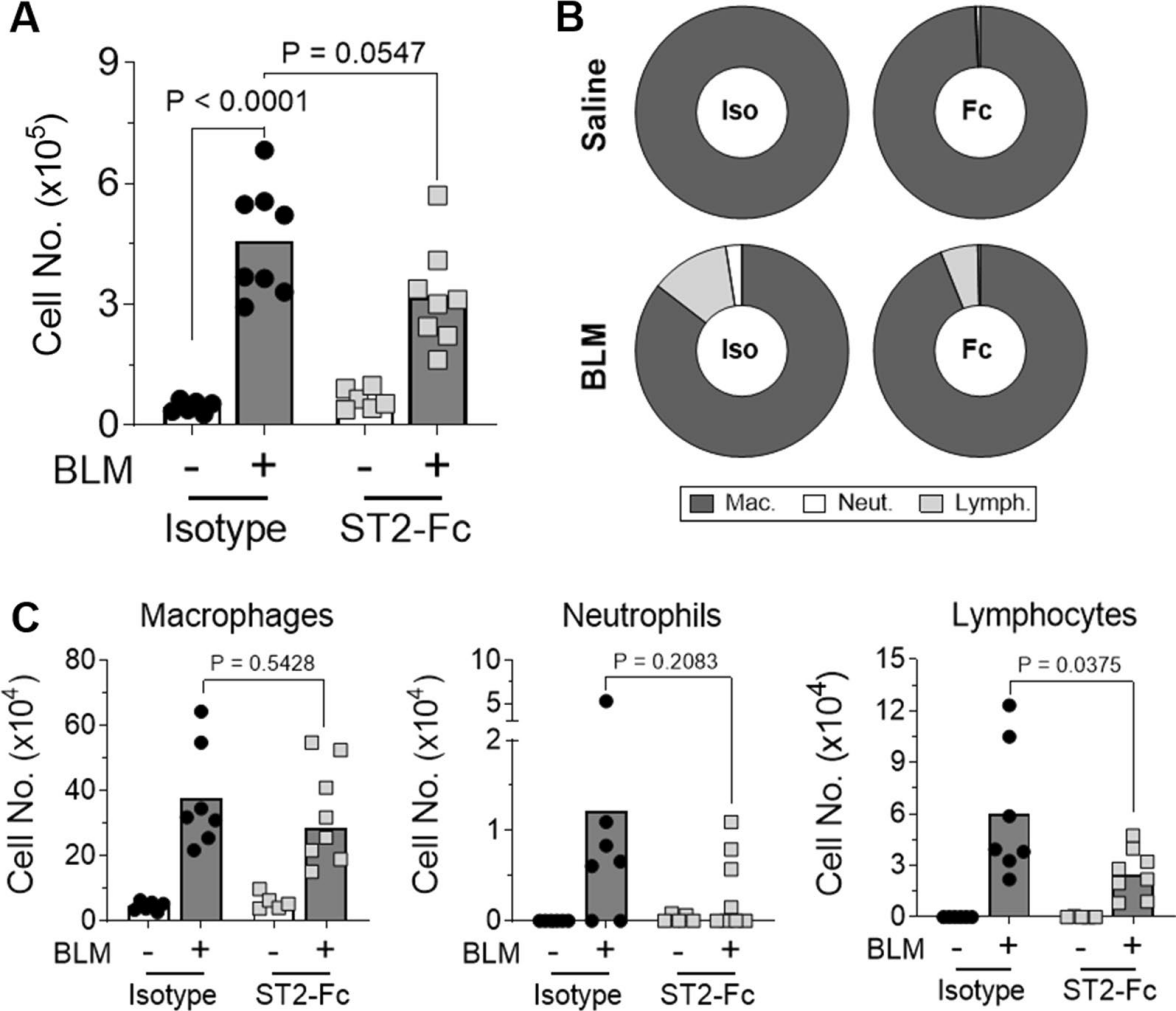
The effects of the ST2-Fc fusion protein on BLM-induced inflammation. Total BAL cell counts were performed (**A**) and the percentage (**B**) and number (**C**) of macrophages, neutrophils and lymphocytes calculated following cytospin analysis. Statistical analysis was performed by Two-way ANOVA with Tukey’s multiple comparisons test (**A**) or Unpaired T-test (**B, C**). For **A** and **C**, bars indicate mean values and each data point represents a single mouse. For Figure **B** percentages are displayed as mean values. For all Figures, n = 6–8 per treatment group

Following BLM administration, total BAL cell numbers were significantly higher than in saline treated lungs in the isotype control animals. Treatment with ST2-Fc did not result in a substantial reduction in the total number of cells in the BAL of BLM treated mice (Fig. 6A). However, BLM mice exposed to ST2-Fc showed a significant reduction in the percentage (Fig. 6B) and number (Fig. 6C) of lymphocytes in the BAL and a trend towards a reduction in the number of neutrophils compared with isotype control, suggesting the ST2-Fc fusion protein penetrated the lung and engaged its target mechanism.

### IL-33 does not induce pro-fibrotic changes in human PCLS

**Fig. 7:**
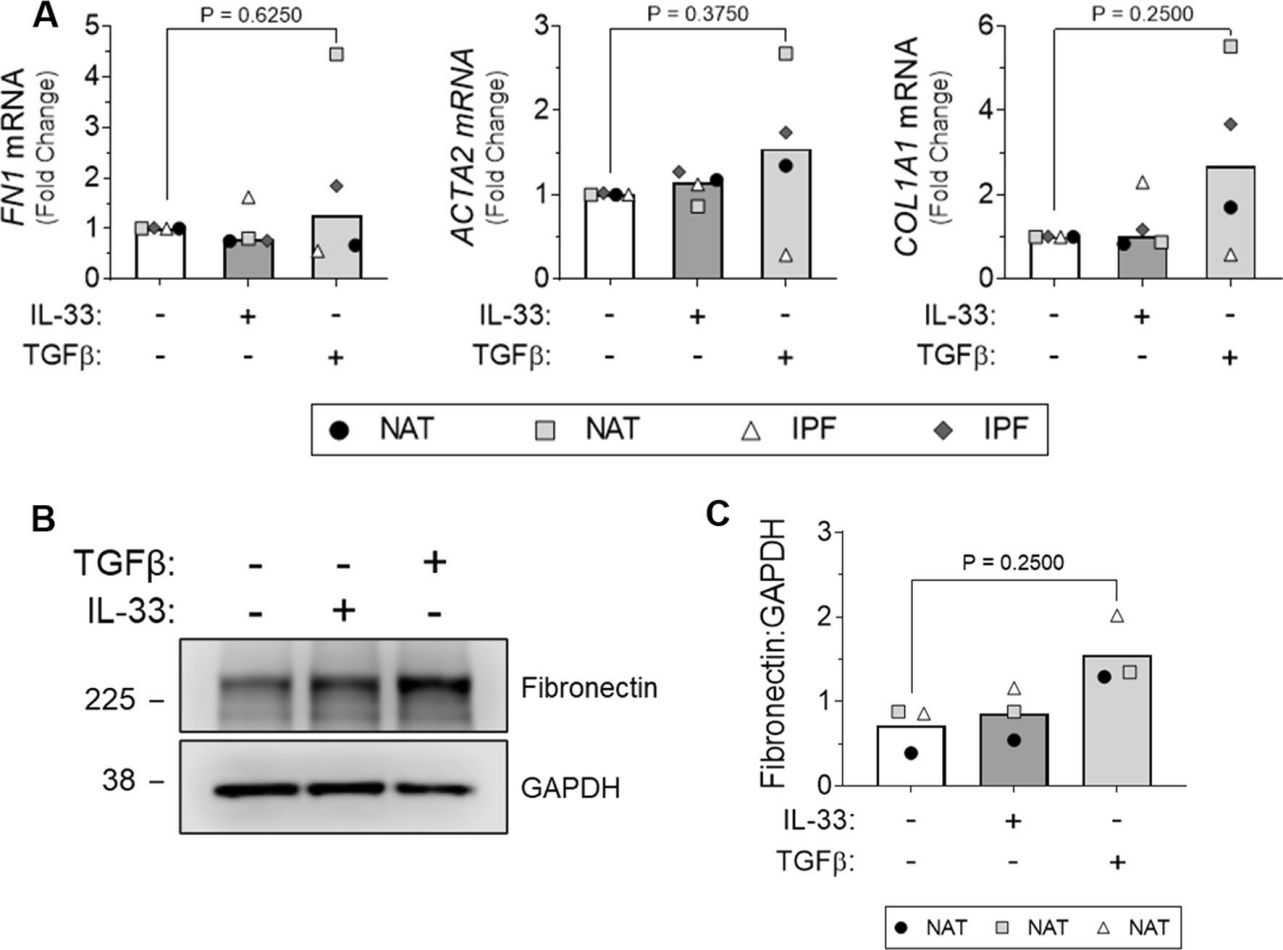
The effect of IL-33 treatment on fibrotic marker expression by human PCLS. PCLS from normal adjacent (NAT) and IPF lung tissue were stimulated with 30 ng/ml of IL-33 or 2 ng/ml of TGFβ. *FN1*, *ACTA2* and *COL1A1* mRNA expression by NAT and IPF PCLS was measured by qPCR and normalised to the unstimulated media alone control for each individual donor (**A**). Fibronectin expression was assessed by western blot (10 μg total protein/lane) following SDS-PAGE analysis (**B**) and quantified relative to GAPDH via densitometry (**C**). A representative cropped western blot from a single donor is shown. For Figures **A** & **C** bars represent median values and each data point represents an independent donor. n = 3–4. Statistical analysis performed by Wilcoxon signed-rank test

Finally, to investigate the pro-fibrotic potential of extracellular IL-33 in IPF, PCLS from normal adjacent tissue (NAT) and IPF lung were stimulated with IL-33 or TGFβ and changes in gene expression and protein levels of fibrotic markers assessed. IL-33 had no effect on the expression of *FN1*, *ACTA2* and *COL1A1* gene expression (Fig. 7A) with a similar trend observed for fibronectin at the protein level (Fig. 7B, C). In contrast, non-significant increases in *COL1A1* mRNA (Fig. 7A) and fibronectin protein levels (Fig. 7C) were apparent with TGFβ treatment suggesting that the PCLS were capable of responding to fibrotic stimuli. As stimulation with exogenous IL-33 failed to induce fibrotic changes in our PCLS, we suspect the effects of TGFβ on *COL1A1* mRNA and fibronectin protein are IL-33-independent.

## Discussion

IPF is a debilitating interstitial lung disease with a poor prognosis and limited treatment options. Despite recent work suggesting that IL-33 may play an important fibrogenic role in IPF [11–19], the use of prophylactic dosing regimens in all previous studies means that the therapeutic benefit of targeting IL-33:ST2 signalling in IPF is poorly understood. Here we show that despite being expressed in fibroblasts and upregulated by treatment with the potent pro-fibrotic cytokine TGFβ, extracellular IL-33 has no direct effect on the pro-fibrotic activity of these cells. In addition, using the BLM mouse model of lung fibrosis, we demonstrate that therapeutic dosing with an ST2-Fc fusion protein has no effect on the severity of established BLM-induced fibrosis. Furthermore, stimulation with IL-33 does not induce pro-fibrotic changes in human PCLS. Considered together, these findings suggest that the IL-33:ST2 axis is unlikely to play a central fibrogenic role in IPF.

Due to their role as one of the key effector cell types during the development of IPF [1], the expression of IL-33 by fibroblasts in ILD lung sections was assessed by IHC. Although we cannot make definitive conclusions in the absence of cell-type specific markers, the spindle-like morphology of some IL-33 positive cells, our IL-33 gene and protein data in IPF HLFs and the reported expression of IL-33 by freshly isolated [35] and cultured [14] IPF fibroblasts, collectively suggest that a proportion of the IL-33 positive cells in our ILD lung sections were fibroblasts.

In agreement with our IHC data, IPF HLFs were found to express IL-33 although in contrast with a previous report we detected no difference in expression between non-IPF and IPF cells maintained in culture [14]. These data are supported by single-cell RNA sequencing data from the IPF cell atlas [35] which indicates that IL-33 is not overexpressed in interstitial lung fibroblasts from IPF patients. Although there is no clear explanation for the different patterns of IL-33 expression in non-IPF and IPF fibroblasts in our study and that of Luzina and colleagues, it may reflect differences in culture conditions and their inclusion of data from scleroderma patients [14].

To determine if IL-33 expression by fibroblasts could be regulated by the pro-fibrotic microenvironment reported in the lungs of IPF patients, HLFs were stimulated with TGFβ. Interestingly, TGFβ increased IL-33 expression by HLFs in our study. As TGFβ has been previously shown to decrease IL-33 expression by smooth muscle cells [36] and alveolar epithelial cells [37], our results suggest that TGFβ-induced IL-33 expression is cell type specific. Moreover, given the increased numbers of fibroblasts in IPF tissue [1], the elevated levels of TGFβ [38], and the ability of this cytokine to drive epithelial to mesenchymal transition [39], these findings may explain the higher expression of IL-33 reported in the lung tissue of IPF patients [13, 14].

Although cell-associated IL-33 protein levels returned to baseline 24 h after stimulation, no secreted IL-33 could be measured in supernatants collected from TGFβ stimulated HLFs. As cell death by necrosis [5], mechanical injury [4], or viral infection [6] is required for IL-33 release from other cell types, it is possible that excess TGFβ-induced IL-33 is degraded in the proteasome [40] of HLFs rather than being released. However, as IL-33 release in the absence of cell death has also been reported [41, 42], it is also possible that IL-33 is released from TGFβ treated HLFs but at a level below the lower limit of detection of the assay used [43].

In the majority of our western blots assessing IL-33 expression by HLFs, a band at 22/23 kDa was detected in addition to the expected band at 31 kDa. As the full-length 31 kDa protein can be processed by cell-derived proteases [44], it is possible that this band represents a cleaved form of IL-33. Based on reports from Scott et al. [43] using a similar protein extraction method, we suspect the 22/23 kDa band is likely an artefact of mechanical cell lysis. As we are uncertain of the biological relevance of this band in our experiments, only bands at 31 kDa have been labelled and quantified as IL-33.

To assess whether IL-33 secreted either from fibroblasts themselves or from other cell types within the lung could mediate pro-fibrotic effects on fibroblasts, we stimulated non-IPF and IPF HLFs with exogenous IL-33 and found no evidence of increased expression of pro-inflammatory or pro-fibrotic genes suggesting that this cytokine does not promote fibrogenesis. Although studies assessing human [45] and murine cells [46] have demonstrated fibroblast responsiveness to IL-33, our results are consistent with those of Yagami and colleagues [47] and suggest that extracellular IL-33 cannot directly increase the pro-fibrotic activity of fibroblasts during the development of IPF as they lack the IL-33 receptor ST2.

Given the predominantly nuclear pattern of IL-33 expression in IPF lung tissue, it is possible that TGFβ-induced IL-33 has intracellular rather than extracellular pro-fibrotic effects on HLFs. However, a recent report by Luzina et al. determined that although overexpression of nuclear IL-33 in fibroblasts resulted in phosphorylation of SMAD3, it did not induce collagen gene transcription and actually attenuated TGF-β-induced levels of collagen I and III mRNAs [48]. Additionally, using human fibroblasts and an in vivo model of unilateral ureteral obstruction, Gatti et al. recently suggested a novel role for nuclear IL-33 as a repressor of interstitial cell extracellular matrix deposition rather than as a mediator of fibrosis [49]. As comprehensive IL-33 knockdown failed to regulate the expression of any proteins other than IL-33 by endothelial cells [50] and deletion of the nuclear localisation signal of IL-33 resulted in a fatal hyperinflammatory response in vivo [51], it is possible that the nuclear pattern of IL-33 expression observed in fibrotic lung tissue may reflect the importance of nuclear storage in controlling the extracellular effects of IL-33 instead of an intracellular, fibrogenic role in transcription.

Consistent with previous studies using ST2 knockout mice [16, 18], IL-33 neutralising antibodies [16], and soluble ST2 [17], treatment with the ST2-Fc fusion protein reduced the number of lymphocytes and neutrophils in the BAL of BLM treated mice. As adenoviral overexpression [13, 14] and intranasal delivery of recombinant IL-33 [16] has been reported to increase lymphocyte and neutrophil numbers, our results, in combination with previously published data, suggest that IL-33 signalling promotes inflammation in the early injury phase of the BLM model of lung fibrosis.

In contrast with previous studies targeting IL-33:ST2 signalling [16–19], the ST2-Fc fusion protein failed to reduce BLM-induced fibrosis. Considering IL-33 is a potent pro-inflammatory cytokine [9], the reduced fibrogenesis reported in all previous studies likely reflects a reduction in the inflammatory response required for the development of BLM-induced fibrosis, rather than a true anti-fibrotic effect. Indeed, as all previous studies in the BLM mouse model have blocked IL-33 signalling prior to or during the inflammatory phase [16–19] we believe that our therapeutic dosing approach, intervening only during the fibrotic phase, explains why our findings differ from other reported studies. Furthermore, since IL-33 had no direct effect on HLFs and failed to induce pro-fibrotic changes in human PCLS, we propose that the IL-33:ST2 axis in isolation is unlikely to play a key pro-fibrotic role in IPF.

## Conclusions

In conclusion, IL-33 has no ST2-dependent effects on human lung fibroblasts in vitro nor does it act as a major fibrotic mediator in either the BLM mouse model of pulmonary fibrosis or human PCLS. Although our data do not support a role for inhibiting the IL-33:ST2 axis as a treatment for established pulmonary fibrosis, therapeutic targeting of this pathway in patients with inflammatory complications of IPF, such as acute exacerbations following viral infection, may prove beneficial and therefore remains an attractive possibility warranting further investigation.

## Supplementary Information

**Additional file 1. Figure S1.** Whole IL-33 western blot for Fig. 1F. Basal IL-33 protein expression by non-IPF (n=3) and IPF (n=4) HLFs assessed by western blot. 20 µg protein/lane loaded for HLF and HUVEC (HUV.) lysates. α-Tubulin was used as a loading control. **Figure S2.** Whole IL-33 western blot for Fig. 2B. HLFs from a representative non-IPF donor were stimulated with 2 ng/ml TGFβ for 8 and 24 h. 20 μg protein/lane was separated by SDS-PAGE and IL-33 expression assessed by western blot. α-Tubulin was used as a loading control. **Figure S3.** Whole IL-33 western blot for Fig. 2C. HLFs from a representative IPF donor were stimulated with 2 ng/ml TGFβ for 4, 8 and 24 h. 20 μg protein/lane was separated by SDS-PAGE and IL-33 expression assessed by western blot. α-Tubulin was used as a loading control

## Data Availability

The datasets generated during and/or analysed during the current study are available from the corresponding author on reasonable request.

## Abbreviations

ARRIVE: Animal Research: Reporting of In Vivo Experiments
BAL: Bronchoalveolar lavage
BALF: Bronchoalveolar lavage fluid
BLM: Bleomycin
HLFs: Human lung fibroblasts
HPAECs: Human pulmonary artery endothelial cells
HUVECs: Human umbilical vein endothelial cells
HYP.: Hydroxyproline
i.p.: Intraperitoneal
IHC: Immunohistochemistry
ILD: Interstitial lung disease
IL1RL1: Interleukin-1 receptor like 1
IL-33: Interleukin-33
IPF: Idiopathic pulmonary fibrosis
MDMs: Monocyte-derived macrophages
NAT: Normal adjacent lung tissue
PCLS: Precision-cut lung slices
qPCR: Quantitative real-time polymerase chain reaction
sST2: Soluble ST2
ST2: Serum stimulated-2
TGFβ: Transforming growth factor-β
