# Supplementary material for: The IL-33:ST2 axis is unlikely to play a central fibrogenic role in idiopathic pulmonary fibrosis
Source: Respir Res. 2023 Mar 23;24:89. doi: 10.1186/s12931-023-02334-4 (PMC10035257; doi:10.1186/s12931-023-02334-4)

**Additional file 1: Figures S1–S3**

**Additional file 1: Figure S1: Whole IL-33 western blot for Figure 1F.** Basal IL-33 protein expression by non-IPF (n=3) and IPF (n=4) HLFs assessed by western blot. 20 µg protein/lane loaded for HLF and HUVEC (HUV.) lysates. α-Tubulin was used as a loading control.


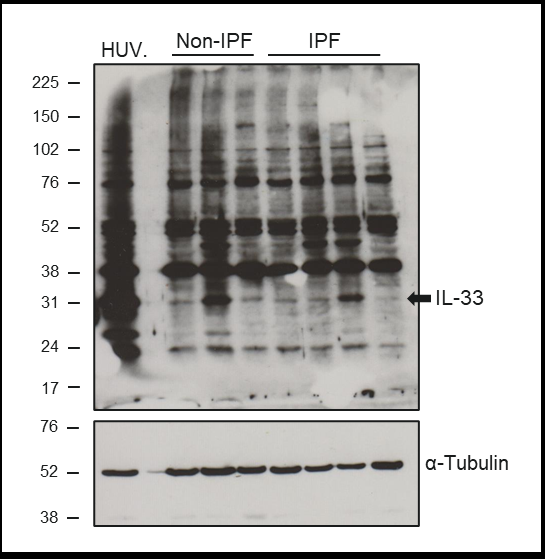


**Additional file 1: Figure S2: Whole IL-33 western blot for Figure 2B.** HLFs from a representative non-IPF donor were stimulated with 2 ng/ml TGFβ for 8 and 24 hours**.** 20 μg protein/lane was separated by SDS-PAGE and IL-33 expression assessed by western blot. α-Tubulin was used as a loading control.


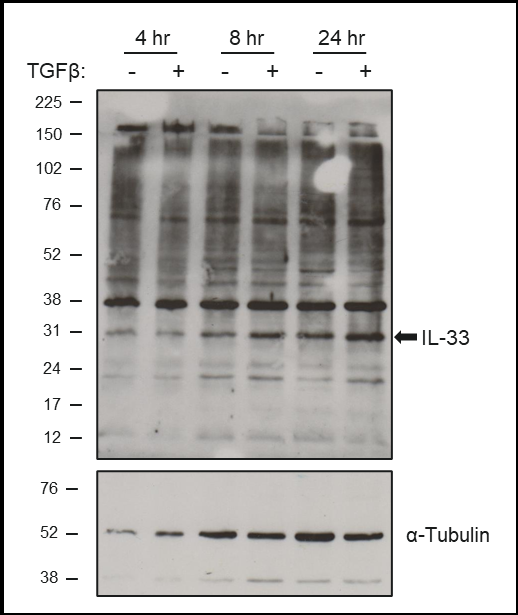


**Additional file 1: Figure S3:** **Whole IL-33 western blot for Figure 2C.** HLFs from a representative IPF donor were stimulated with 2 ng/ml TGFβ for 4, 8 and 24 hours**.** 20 μg protein/lane was separated by SDS-PAGE and IL-33 expression assessed by western blot. α-Tubulin was used as a loading control.


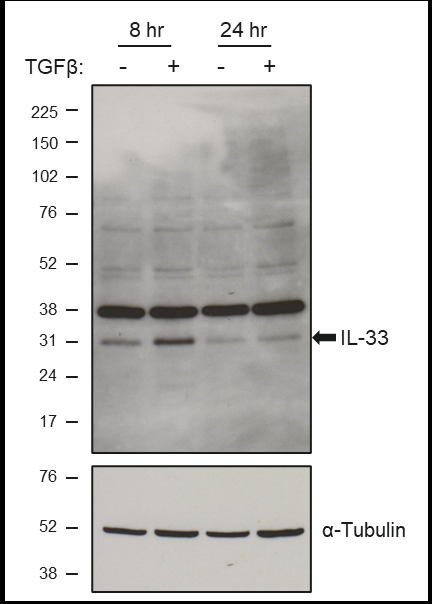

Supplement: Supplementary file 1 — Additional file 1. Figure S1. Whole IL-33 western blot for Fig. 1F. Basal IL-33 protein expression by non-IPF (n=3) and IPF (n=4) HLFs assessed by western blot. 20 µg protein/lane loaded for HLF and HUVEC (HUV.) lysates. α-Tubulin was used as a loading control. Figure S2. Whole IL-33 western blot for Fig. 2B. HLFs from a representative non-IPF donor were stimulated with 2 ng/ml TGFβ for 8 and 24 h. 20 μg protein/lane was separated by SDS-PAGE and IL-33 expression assessed by western blot. α-Tubulin was used as a loading control. Figure S3. Whole IL-33 western blot for Fig. 2C. HLFs from a representative IPF donor were stimulated with 2 ng/ml TGFβ for 4, 8 and 24 h. 20 μg protein/lane was separated by SDS-PAGE and IL-33 expression assessed by western blot. α-Tubulin was used as a loading control [file 12931_2023_2334_MOESM1_ESM.docx]
